# Perspectives of surface plasmon resonance sensors for optimized biogas methanation

**DOI:** 10.1002/elsc.201900063

**Published:** 2019-09-05

**Authors:** Jacob J. Lamb, Olivier Bernard, Shiplu Sarker, Kristian M. Lien, Dag Roar Hjelme

**Affiliations:** ^1^ Department of Electronic Systems & ENERSENSE NTNU Trondheim Norway; ^2^ Department of Energy and Process Engineering & ENERSENSE NTNU Trondheim Norway; ^3^ BIOCORE, Biological control of artificial ecosystems Université Côte d'Azur, Inria, INRA Sophia‐Antipolis France; ^4^ Department of Manufacturing and Civil Engineering NTNU Gjøvik Norway

**Keywords:** anaerobic digestion, biogas, hydrogen, methanation, optical sensors, renewable energy, surface plasmon resonance

## Abstract

Biogas production is becoming significantly viable as an energy source for replacing fossil‐based fuels. The further development of the biogas production process could lead to significant improvements in its potential. Wastewater treatment currently accounts for 3% of the electrical energy load in developed countries, while it could be developed to provide a source of nitrogen and phosphorus, in addition to energy. The improvement of anaerobic digestion (AD) detection technologies is the cornerstone to reach higher methane productivities and develop fully automatized processes to decrease operational costs. New sensors are requested to automatically obtain a better interpretation of the complex and dynamical internal reactor environment. This will require detailed systematic detection in order to realize a near‐optimal production process. In this review, optical fiber‐based sensors will be discussed to assess their potential for use in AD. There is currently a disparity between the complexity of AD, and online detection. By improving the durability, sensitivity, and cost of dissolved H_2_ (as well as H_2_S, acetic acid, ammonia, and methane) sensor technology, further understanding of the AD process may allow the prevention of process failure. The emergence of surface plasmon resonance (SPR) sensing with optical fibers coupled with the H_2_‐sensitive metal palladium, allows detection of dissolved hydrogen in liquid. By implementing these SPR sensors into AD, improvements to the biogas production process, even at small scales, may be achieved by guiding the process in the optimum direction, avoiding the collapse of the biological process. This review intends to assess the feasibility of online, cost‐effective, rapid, and efficient detection of dissolved H_2_, as well as briefly assessing H_2_S, acetic acid, ammonia, and methane in AD by SPR.

**Abbreviation**
AD: anaerobic digestion

## 1 INTRODUCTION

One route to production of renewable, clean energy is through biogas production, where biogas is derived from organic waste materials. The production of biogas is achieved through the anaerobic digestion (AD) of the organic waste materials by various microorganisms 1. Moreover, in developing countries, wastewater treatment can be an energy‐requiring process, where up to 3% of the electrical load is required 2. By improving such wastewater treatment systems in developing countries to produce larger quantities of biogas, these energy requiring systems could be turned into energy producing systems that also provide nitrogen and phosphorus for fertilizers 2. The so‐called power‐to‐gas route, where surplus renewable electrical energy is used to produce H_2_ by electrolysis, can explain this. However, H_2_ utilization is facing unsolved bottlenecks related to H_2_ transport and storage. An attractive alternative to H_2_ distribution is to convert H_2_ to methane in a biological methanation process 3, 4, 5, 6, 7. Methane has a much higher volumetric energy content than H_2_ and thus a lower storage transport cost, and can be distributed in existing natural gas infrastructure 8. By utilizing the methanation process, H_2_ can be used to reduce the CO_2_ in the biogas produced 3, 4, 5, 6, 7. A promising approach is to input H_2_ to the biogas reactor such that the methanation takes place within the reactor 6. However, increasing the concentration of dissolved H_2_ in the reactor can inhibit the production of biogas and accumulate VFAs, and in the worst case, cause the whole biological process to collapse 9, 10.

It has been hypothesized that adding hydrogen directly into the anaerobic digester may change microbial community composition promoting hydrogenotrophic methanogenesis pathways 11. This enhances the biological production of methane by 20–40% 6, 12, potentially achieving purity of up to 90% 3, 11, 13, 14 when combined with ex situ upgrading. This allows existing biogas plants to be utilized for H_2_, eliminating the need for hydrogen storage (which can be of safety concern) 15. Despite this, dissolved H_2_ can potentially inhibit production of methane, and in the worst case, cause the whole biological process to collapse 9. Moreover, injection of H_2_ to a level exceeding a 4:1 stoichiometric ratio between H_2_ and CO_2_ tends to deplete CO_2_ resulting in the rise of pH 12, and inhibiting the autotrophic hydrogenotrophic methanogenesis process 16. Furthermore, the oxidation of acetate toward methane production is only thermodynamically favorable when the partial pressure of H_2_ is below 74 Pa 17, 18. Therefore, dissolved H_2_ must be monitored continually, especially if H_2_ is directly injected into the anaerobic digester for methane upgradation.

Due to the complexity of the biogas production process, it is not always straightforward to determine the state of the AD process from measuring the chemical variables within the digester 1. Despite this, the hydrogen content of produced biogas is a very sensitive indication of imbalance between microbial groups within the digester 19, 20, 21. It has been observed that hydrogen content in biogas can be used as a parameter for maintaining stable operation, where the reactor can be feed to produce maximum methane yields, as long as the hydrogen concentration remains low 22, 23. Although hydrogen is straightforward to measure in the gas phase, dissolved hydrogen in the liquid phase is considered more appropriate as the liquid to gas mass transfer of hydrogen introduces a significant delay in detection 24. Therefore, dissolved H_2_ is recognized as a better approach to an early warning indicator and an excellent complement to VFA monitoring 9. Dissolved H_2_ concentrations are, therefore, required to control parameters for biogas production monitoring and utilizing H_2_ for further methanation of CO_2_ 6, 7. It is essential that dissolved H_2_ be monitored closely to gain an understanding of the current biological process within the digester.

**PRACTICAL APPLICATION**

By improving the technologies associated with variable detection in anaerobic digestion, anaerobic digestion plants will be able to obtain warning indications of possible biological process failure before it occurs. This will prevent significant losses associated with biological process failure and collapse, maintaining optimal performance of the plant.

Cost‐effective sensor technologies for monitoring and analysis of H_2_ within the reactor are crucial for improving efficiency, productivity, and cost of biogas production in many plants. There are many methods for achieving monitoring of a variety of parameters within bioreactors. These may rely on sample extraction, but in some cases, the sensor can be interfaced directly with the internal environment of the bioreactor. Despite this, there are still improvements required to make ideal sensors for the measurement of dissolved H_2_ within the biogas production process. Ideally, a monitoring system must have cost‐effective, accurate, specific, stable, and sensitive sensors, which can send data to software programs for analysis. This data can then be computationally analyzed, allowing the correlation of the sensor data to a specific parameter or process model. Furthermore, with the increase in small‐scale plants, there is a significant demand for sensor technologies that do not have an associated high cost.

In this review, contemporary sensor technologies that can combine AD with online sensor technologies focusing on dissolved H_2_ are discussed and compared to current industry methods. The contemporary technologies presented have the potential for improving the efficiency, economic costs, and productivity of biogas plants, as well as allowing online dissolved H_2_ monitoring to facilitate H_2_ injection for methane upgradation. A further brief discussion will assess H_2_S, acetic acid, ammonia, and methane surface plasmon resonance (SPR) sensing potential.

## 2 REQUIREMENTS FOR VARIABLE DETECTION DURING BIOGAS METHANATION

There are distinctive ways that sensors can be fused into the AD plant to satisfy the online observing prerequisite. The sensors utilized for estimations of factors inside an AD plant can require the extraction of digestate from the bioreactor, filtration and external sampling (at‐line sensor), the sensor to be located inside the digester (in‐line sensor), or to be removed from the digester, and sampled in a lab (disconnected sensor) 25.

The in‐line sensors must be impervious to pressure (somewhat above atmospheric pressure) and temperature (somewhere in the range of 35 and 55°C), as well as having the capacity to be cleaned. Also, they must be significantly impervious to obstruction by fouling. Ideal sensors must provide proper detection quality, having high precision, specificity, and sensitivity. The collected information must then automatically be processed and used in the supervision system. The estimations should deliver consistency and steadiness despite changes in medium, biological, or chemical contents. In practice, the benefits of the sensor in terms of increasing process reliability and efficiency must be compared to its price, including manpower requirement for human intervention (setup, calibration, and cleaning).

For live, online detection of an AD plant to be valuable, the reaction time of the sensors is a critical feature 25. Even though the delay of measurements is explicit to every sensor and application, the time required to acquire an outcome must be small (<10 min) concerning the biological dynamics within the digester. Moreover, with regard to H_2_ detection, measurements in the liquid phase allow earlier detection when compared to measurements in the gas phase, avoiding bias measurements. This is due to the relatively slow mass transfer of dissolved H_2_ from the liquid phase to the gas phase 6, 22. More explicitly, the time required to acquire a sensor outcome will depend altogether on the retention time of the AD reactor, duration of analysis, and the dead volume of the filtration framework utilized. In addition, it must be a lot quicker than the time required for the gathering of H_2_ to happen to distinguish and avoid a stress event. With this in mind, the sensor framework must be tailored to the procedure with satisfactory sampling locations. Otherwise, the low effectiveness of the process control will occur 26.

The location of the sensor inside the digester is fundamental, as the digester can be homogeneous or inhomogeneous. This depends mainly on the feedstock type, process size, and AD technologies. In addition, there are AD methods that have intended inhomogeneous chemical gradients inside the reactor (e.g. up‐flow anaerobic sludge blanket (UASB) and fluidized bed reactors), and this is exacerbated with the expansion of reactor size. If the digester is significantly inhomogeneous, several sensors might be required to decipher the state of the digester completely.

There are three primary phases (solid, liquid, and gas) inside an AD plant. These phases have diverse properties, factors, and prerequisites when considering in‐line sensors. The liquid phase of the anaerobic digester is an intricate blend of different microorganisms, substrates, nutrients, products, metabolites, and dissolved gases. Because of the diffusion rate of components from liquid to gas, it is attractive to make estimations specifically from the liquid phase of the digester. This can decrease the lag time between changes in the AD biological dynamics and identification of these changes.

## 3 CURRENT METHODS OF H_2_ DETECTION IN AD

### 3.1 Dissolved H_2_ detection

Dissolved H_2_ produced in AD has long been recognized as an early warning indicator and an excellent complement to VFA monitoring 9, 24. Additionally, the in situ hydrogen injection into the anaerobic digester is a promising process aimed at increasing the biogas methane content, at the expense of the CO_2_ content.

**Figure 1:**
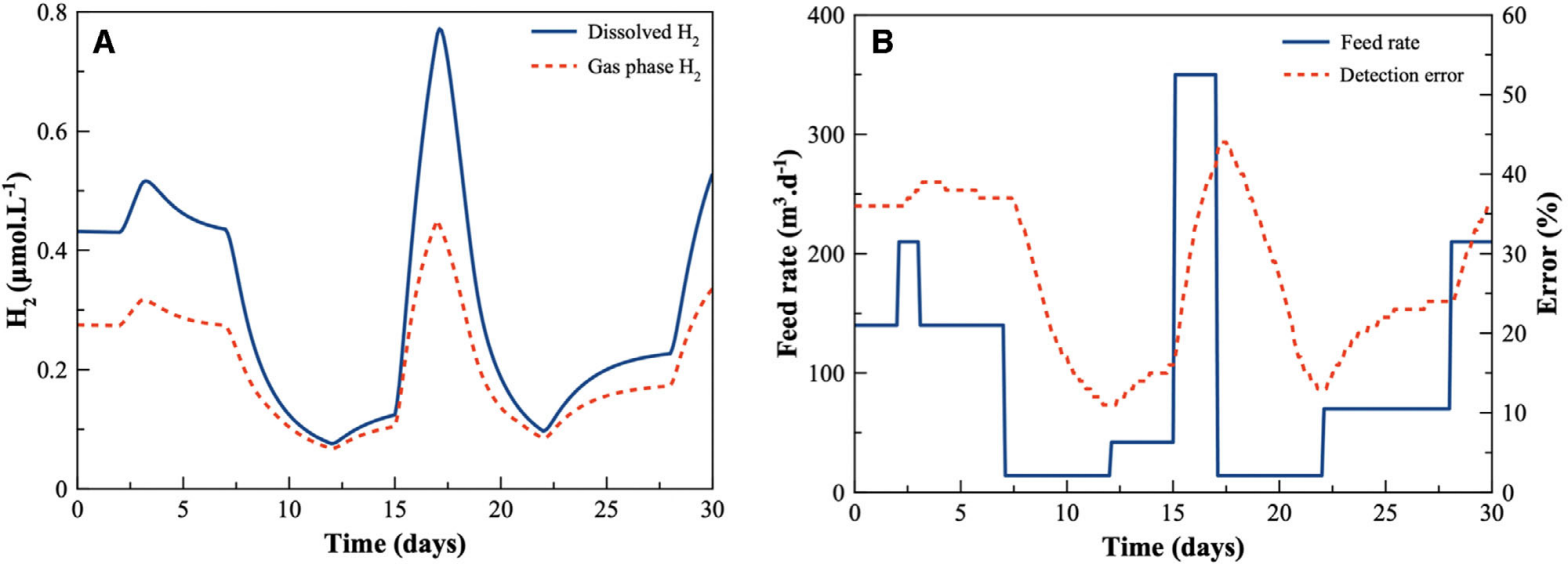
A computational model of detection bias. (A) The concentration of dissolved H_2_ in the liquid phase of an anaerobic digester over a 30 day period (solid blue line) is shown compared to the detection of H_2_ in the gas phase (red dashed line) of the same reactor. (B) The feed rate of the reactor throughout 30 days (solid blue line) is shown with the percentage error between the detected H_2_ and the actual dissolved H_2_ (red dashed line)

Since the critical concentration of dissolved H_2_ is only 74 Pa 17, 18 and difficult to measure, the concentration of dissolved H_2_ in anaerobic biogas production is currently calculated from a gas fraction of H_2_ detected in the gas phase of the digester. However, due to the limited H_2_ mass‐transfer coefficient, there is a significant time delay between an increase in H_2_ concentration in the liquid and an increase in the gas phase of the digester. Using the ADM1 model, an assessment was made of the hydrogenotrophic activity in reaction to a dynamic feeding regime. A 1500 m^3^ reactor with 1400 m^3^ liquid phase and 100 m^3^ gas phase was simulated. The difference between actual dissolved H_2_ in the liquid phase, and the measured H_2_ in the gas phase as determined using the ADM1, as shown in Figure 1, is significant. Furthermore, this difference (error) between the true (dissolved) and the measured (gas phase) concentrations of H_2_ was observed to be over 40% at times. This shows that such errors and time delays represent a severe limitation for optimal process control, producing a significant bias 9, 24.

Extraction techniques designed for off‐line dissolved H_2_ monitoring are time consuming and potentially introduce a bias due to outgassing of H_2_, and, therefore, not an option for the rapid process control desired. Other techniques based on the transfer of H_2_ from the liquid through a gas‐permeable membrane followed by gas phase quantification (GC or MS) are considered too sophisticated and expensive for online monitoring. Therefore, there is a definite need for an effective, reliable, and cost‐effective sensor for continuous online monitoring of dissolved H_2_ in anaerobic bioprocesses.

Extraction techniques designed for off‐line dissolved H_2_ monitoring are time consuming and potentially introduce a bias due to outgassing of H_2_, and, therefore, not an option for the rapid process control desired. Other techniques based on the transfer of H_2_ from the liquid through a gas‐permeable membrane followed by gas phase quantification (GC or MS) are considered too sophisticated and expensive for online monitoring. Therefore, there is a definite need for an effective, reliable, and cost‐effective sensor for continuous online monitoring of dissolved H_2_ in anaerobic bioprocesses.

### 3.2 Gas‐phase detection

Concentrations of H_2_ gas in the headspace of the anaerobic digester is straightforward to measure but is delayed significantly due to the mass‐transfer coefficient of H_2_ and can result in significantly bias measurements with a large error (Figure 1). Therefore, the main drawback of the detection in the headspace is that sudden changes in dissolved H_2_ levels will not be detected for some time, and, therefore, process failure could occur before any signals of an H_2_ increase are detected. Despite this, membrane‐covered Clark‐type electrodes can be adapted to measure H_2_ in the headspace of the reactor, with negligible biofouling of the membrane occurring when in gas phase. Thermal conductivity sensors can also gauge the concentration of H_2_ gas in the headspace. These sensors use a small heated filament to detect thermal conductivity of the gases in simple gas mixtures, allowing the determination of the percentage of specific gases of interest within a mixture.

Palladium metal oxide semiconductor sensors have also been studied for their suitability for H_2_ detection in the gas phase of AD 27. The palladium can become palladium hydride in the presence of H_2_, a rapid and reversible reaction that can alter the conductivity of the metal and used to determine the ppm of H_2_. Alternatively, GC instruments equipped with thermal conductivity detectors can be used to determine H_2_ 21. GC is a very accurate method of H_2_ detection in the gas phase and is used as a reference measurement technique for all other H_2_ detection methods.

### 3.3 Liquid‐phase detection

Hydrogen can be detected using a membrane‐covered Clark‐type electrode 28 in the liquid phase as well. There are extraction and H_2_ permeable membrane methods designed for dissolved H_2_ monitoring within the digester, but these can be expensive and time‐consuming processes. The membrane requiring Clark‐type electrodes can be used for online monitoring of dissolved H_2_ in the liquid phase, which relies on the oxidation of H_2_ at a Pt electrode and dissociation of molecular hydrogen at a Pd electrode 29, 30. Unfortunately, these are prone to biofouling on the membrane surface. Once biofouling occurs, the measurements from this type of electrode are mostly unreliable, and therefore, the membrane must regularly be replaced to avoid this. So far all reported electrochemical sensors for H_2_ detection suffer from membrane fouling resulting in a short lifetime (around 2 days), low selectivity, and low S/N 30. Although these Clark‐type sensors can operate at room temperature, temperature fluctuations must also be taken into account to maintain correct O_2_ and H_2_ calculations 31.

Measurements of dissolved hydrogen can also be performed using hydrogen microsensors. Microsensors allow H_2_ to diffuse from the internal liquid phase of the anaerobic digester through a silicone membrane to a platinum anode that is polarized against an internal reference. The current between the palladium electrode and the reference is linearly indicative of the dissolved hydrogen at the tip of the sensor. The current change is in the picoamp range and is measured using a picoammeter. An alternative method for dissolved H_2_ detection was described by Björnsson et al. 29. Here, a liquid‐to‐gas membrane was utilized to extract dissolved H_2_ from the liquid phase, through a Teflon membrane placed in the internal environment of the anaerobic digester. The H_2_ was then detected using a palladium metal oxide semiconductor sensor 29.

### 3.4 Remarks

The methods outlined here suffer from disadvantages including sample preparation, contamination, and biofouling. GC is a real proven alternative at very high accuracy, but requires advanced systems for auto‐sampling, transfer of the sample and filtering, avoidance of outgassing, and requires a significant amount of time to obtain a measurement. Forms of ultrafiltration are required for liquid phase detection to produce a clear particle‐free sample for GC and electrochemical detection methods. This filtration will result in biofouling in the long term, a property that must be considered when using such methods for detection in the liquid phase.

Measurements undertaken in the gas phase of the reactor have limited issues with biofouling of filtration systems; however, the presence of H_2_S in the biogas can reduce the sensitivity of the sensor systems 27.

## 4 EMERGING SPR SENSOR TECHNOLOGIES FOR AD

More recently, various optical sensor principles have been proposed for H_2_ monitoring 44, 45, 46, 47, 48, 49, 50, 51, 52, 53, 54, 55, 56. Sensors based on the H_2_ uptake in palladium appear particularly promising. The volume expansion of palladium films or nanoparticles caused by metal‐hydride formation can be measured very accurately using optical techniques 44, 45, 49, 52, 54, 55. Additionally, the SPR of gold (when coupled to palladium in a layer stack), can be measured accurately, with the plasmon resonance frequency being modulated by the hydrogenation of the palladium layer 48, 50, 53.

More recently, various optical sensor principles have been proposed for H_2_ monitoring 44, 45, 46, 47, 48, 49, 50, 51, 52, 53, 54, 55, 56. Sensors based on the H_2_ uptake in palladium appear particularly promising. The volume expansion of palladium films or nanoparticles caused by metal‐hydride formation can be measured very accurately using optical techniques 44, 45, 49, 52, 54, 55. Additionally, the SPR of gold (when coupled to palladium in a layer stack), can be measured accurately, with the plasmon resonance frequency being modulated by the hydrogenation of the palladium layer 48, 50, 53.

**Figure 2:**
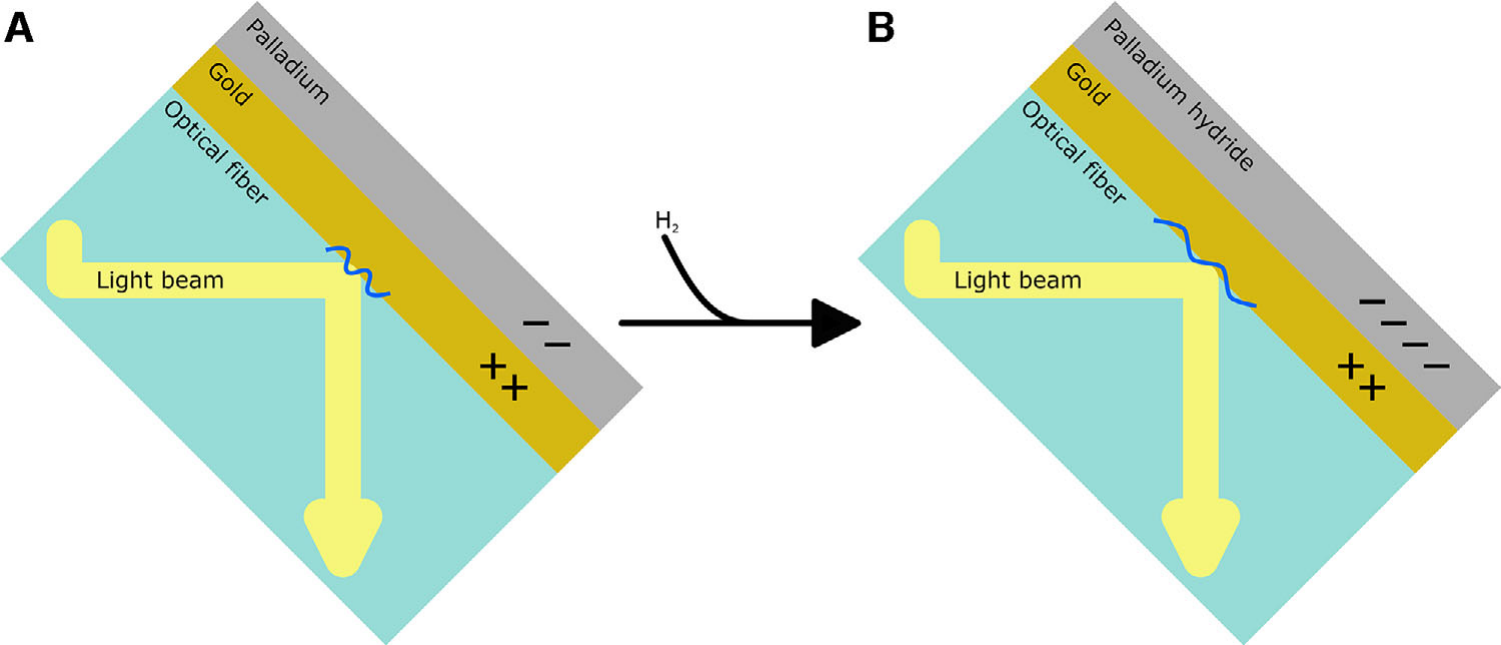
Optical sensor technologies using surface plasmon resonance. The oscillation of surface electrons of gold will interact with waveforms of light that oscillate at the same frequency. The oscillation frequency of these surface electrons can be altered by the relative dielectric constant between gold (positive permittivity material), and another material close by (in this case, palladium, a negative permittivity material). The negative permittivity material can become more negative (in this case, when H_2_ reacts with palladium), causing the oscillation frequency of gold's surface electrons to change as a result of the H_2_ concentration, thus a shift in the wavelength of light that interacts with gold's surface electrons

SPR sensors exploit the interaction of incident electromagnetic waves with the oscillation of the plasmon wave of a material (e.g. gold). When the momentum of incident electromagnetic waves matches the momentum of the surface plasmon waves in the gold, the electromagnetic waves are coupled to the plasmon wave and are diminished (Figure 2). The frequency of the plasmon wave can be altered by changes in the materials used, therefore, changing the wavelength of incident electromagnetic waves absorbed. An example of this is the use of a bare, cladding‐less optical fiber with a gold and palladium layer‐stack. The incident electromagnetic wave interacts with the plasmon wave on the surface of the gold layer, and this plasmon wave can be modulated by changes in the permittivity of the gold.

By adding an H_2_‐sensitive layer of palladium, the formation of palladium hydride effects the electrical field of the gold. This changes the permittivity, resulting in a change of the momentum of the SPR of the gold. The interaction of the incident electromagnetic waves with the gold will be changed, causing different electromagnetic waves to be diminished. Using spectroscopic methods, this will culminate in a shift in apparent absorption of electromagnetic waves because of H_2_ presence.

**Figure 3:**
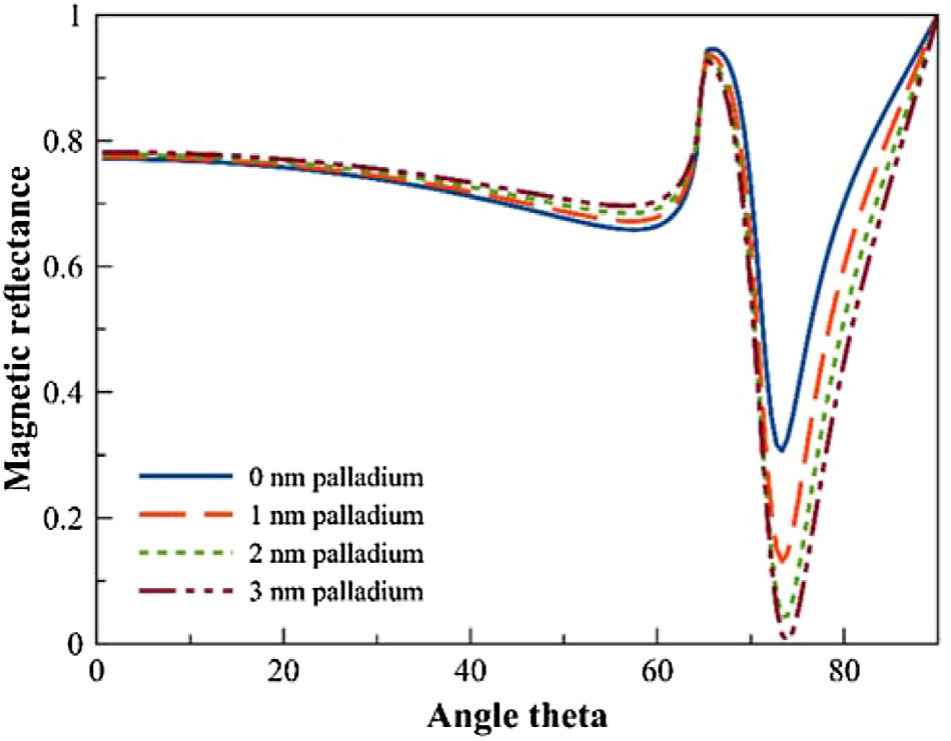
Magnetic reflectance of optical fiber surface boundary. Using the refractive indexes of a silicon optical fiber, gold, palladium, and water, the magnetic reflectance of the 650 nm electromagnetic wave is modeled as a function of angle theta. The thickness of palladium was varied to determine the minimum thickness to achieve a complete reduction in magnetic reflectance

In order to engineer an SPR‐based sensor with palladium as the sensing material for use in the liquid phase of an anaerobic digester, the thicknesses of the gold and palladium layers, and the incident angle of the light to the layers must be considered. Using a straightforward model accounting for the refractive indexes of the silicon optical fiber, gold, palladium, and water, a considerable variation in magnetic reflectance due to the SPR of the optical fiber surface boundary with gold, is observed, as a function of the incident angle and palladium thickness (Figure 3). As the palladium thickness increases, the magnetic reflectance drops at an angle theta of 73.9°, until the magnetic reflectance is completely depleted at a palladium thickness of 3 nm. This observation suggested that to achieve a dissolved H_2_ sensor with high sensitivity and high signal using only gold and palladium metal layers, an incident angle of 73.9°, with thicknesses of 30 nm of gold and 3 nm of palladium is required. Although the thickness of the metal is straightforward to achieve with sputtering deposition techniques, the angle of incidence requires further design considerations.

In order to engineer an SPR‐based sensor with palladium as the sensing material for use in the liquid phase of an anaerobic digester, the thicknesses of the gold and palladium layers, and the incident angle of the light to the layers must be considered. Using a straightforward model accounting for the refractive indexes of the silicon optical fiber, gold, palladium, and water, a considerable variation in magnetic reflectance due to the SPR of the optical fiber surface boundary with gold is observed as a function of the incident angle and palladium thickness (Figure 3). As the palladium thickness increases, the magnetic reflectance drops at an angle theta of 73.9°, until the magnetic reflectance is completely depleted at a palladium thickness of 3 nm. This observation suggested that to achieve a dissolved H_2_ sensor with high sensitivity and high signal using only gold and palladium metal layers, an incident angle of 73.9°, with thicknesses of 30 nm of gold and 3 nm of palladium is required. Although the thickness of the metal is straightforward to achieve with sputtering deposition techniques, the angle of incidence requires further design considerations.

**Figure 4:**
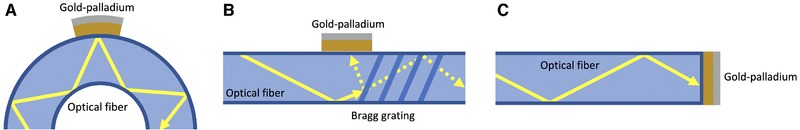
Optical fiber configurations. Optical fibers with a gold‐palladium layer stack showing configurations that can increase the angle of the light incident with the fiber surface. A D‐loop consists of a 180° bend in an optical fiber that results in a higher angle for light interacting with the fiber surface on the outside of the bend (A). Optical fiber Bragg gratings are etched into the core of the fiber, directing light toward the surface of the fiber at the desired angle (B). An endcap allows incident light to interact with a surface at a high incident angle (C)

The metal layers can be deposited on the side surface of the fiber. Increases in the incident light angle can be performed on such designs by placing the optical fiber in a d‐loop (Figure 4A), or by using a Bragg grated fibers (Figure 4B). This will allow the incident light angle to be optimized for coupling of the electromagnetic wave to the SPR wave of the gold layer. Alternatively, the metal layer stack can be deposited on the end‐tip of a fiber, acting as a mirror (Figure 4C). With the use of a multimode fiber, this will allow the electromagnetic wave to achieve coupling with the SPR wave of the gold layer. Furthermore, the metal layer stack could be deposited onto a transparent patch and installed on the internal space of the reactor behind a glass window. Probing of the sensor patch would then be achieved using an external optical fiber, reducing complexity and cost.

### 4.1 Incorporation of software sensors

Information provided by online sensor systems can be extended using mathematical approaches. The two strategies for achieving this are based on methods of data analysis and the representation of the dynamics of the plant by mathematical models. Despite this, to the knowledge of the authors, hydrogen has rarely been estimated with software sensors, most likely due to the lack of tailoring of the existing mathematical models to AD.

#### 4.1.1 Observers

The incorporation of software sensors with online sensor systems allows the prediction of the biological process state by estimating non‐measured variables. In the case of dissolved H_2_ measurements, estimations of the dominating microbial processes within the reactor can be made. Additionally, software sensor systems are a vital supporting component of closed‐loop central strategies. These approaches are also known as state observes or state estimators and have a large amount of theoretical background. Assimilation of the real‐time online measured variable(s) can be combined with the theoretical knowledge base of the AD process through a mathematical model allowing the prediction of variables that have a low frequency or no sampling. Depending on the desired accuracy, sensor information quality, and frequency of sampling, different strategies for the design of an appropriate software sensor can be applied.

Linear frameworks, or extensions thereof, are popular approaches to software sensing. The most known algorithms for achieving software sensors are based on extended Kalman filters and extended Luenberger observers 32. Despite their popularity, the required local linear approximation of the process model reduces the certainties of the stability and convergence properties for wide operating ranges. Furthermore, these algorithms, allowing the approach to be sensitive to any inaccuracy within the model parameters, assume perfect knowledge of the model and its parameters.

Different strategies have been developed by using the mass balance information from the reactor as the base of the estimation scheme to reduce the dependency of the algorithms on parameter uncertainty. Although mass balance only represents the process partially, it can robustly deal with any missing information. Moreover, estimates of kinetics for some processes in the model can be achieved with the support of gaseous flow rates using asymptotic observers 33. These methods can achieve a linear observer by relying on changes of variables cancelling nonlinear terms within the process 34. However, the dilution rate controls the convergence rate when utilizing asymptotic observes. Simultaneous estimating of model parameters and the state of the process can be performed with adaptive observers 35. Alternatively, using the process model and its known parameters allows the tailoring of nonlinear observers with the nonlinearity of the process accounted for by the algorithm 36. A significant limitation of the implementation and calibration of such observer algorithms is their inherent complexity.

Internal observers offer a flexible method by providing guaranteed estimate intervals where the state of the variable lies, avoiding reconstruction of precise numerical values 37, 38. This has been observed successfully for use in AD 39, 40. Due to the uncertainty of concentrations in the influent, this parameter is inherently difficult to use to estimate the process accurately. To overcome this, an algorithm that simultaneously estimates the influent concentrations using both input and state observers has been shown to alleviate this drawback 41. Further development of these observers has resulted in their extension to handle spatial distributions within the anaerobic digester 42, 43.

### 4.2 Calibration of SPR sensors

With using palladium as the sensing material, the selectivity for hydrogen is very high. Therefore, calibration can be achieved straightforwardly by use of GC measurements of hydrogen concentration in the liquid phase.

Different SPR sensor datasets must be comparable to establish successful data analysis. A variety of preprocessing methods can be applied to the data sets to achieve this. Some examples include deviation 57, filtering 58, normalization, standardization, centering, weighing, and scaling 59 of the data sets. Preprocessing is a powerful and sensitive component in data analysis 60, where consideration of the chosen method is required depending on the type of data used. The exclusion of irrelevant data is also a preprocessing method that is essential. This can be achieved by normalization or baseline subtraction/correction. Matching of the data set to known dissolved H_2_ concentrations determined by GC can then be performed.

### 4.3 Deeper insight with SPR sensors

SPR sensors are advantageous as they can also be applied to other variables revealing the internal state of the complex anaerobic process. Acetic acid is a VFA that can be produced during certain stages within the AD of biomass. At high concentrations, it will inhibit the activity of many microorganisms within the digester 9, 10. Using SPR technologies, measurements can be made of acetic acid with the receptor dye aluminium phthalocyanine chloride deposited on a layer of gold 61. This design has been shown to sense acetic acid in a liquid environment 61, suggesting its adaptation into AD may provide an accurate only measurement of acetic acid.

By utilizing colloidal copper nanoparticles, detection of ammonia concentrations have been observed utilizing SPR 62. By suspending colloidal copper nanoparticles in a solgel on a transparent disk behind an observation window within the digester, direct measurements of ammonia levels would be possible by external probing of the sensor disk with an optical fiber.

Methane concentration is usually measured in the gas phase of the digester; however, it is also possible to measure this variable within the liquid phase of the digester. By depositing a layer of modified polydimethylsiloxane (PDMS) polymer that contains cryptophane‐A molecules as the receptor, on top of a layer of gold, methane may be detected using SPR 63. This detection is due to the selective, yet reversible, affinity of cryptophane‐A to methane.

The H_2_S must be removed from the exhaust gases by bio‐filtration methods. It is imperative that the post‐filtration gas has no H_2_S component, and therefore, this gas must be monitored continuously. Titanium oxide is known to react with H_2_S, producing titanium sulphide. This reaction has been observed to cause a change in the resonance frequency of the surface electrons of silver 64. The resonance can then interfere with incident light, as a function of the H_2_S concentration in the gas.

Although these variables have been detected using developed surface plasmon detection techniques, they are all yet to be adapted for utilization in an anaerobic digester.

### 4.4 Perspective and challenges for the developments of SPR sensors

The main limitations for palladium‐based SPR sensors detecting dissolved H_2_ in an AD are palladium poisoning by H_2_S, biofouling and the chemical complexity of the digestate. Moreover, the repeatability of the data from the sensor in a specific digester may change because of influent changes, and the comparability between different digesters with different feedstocks may be a challenge. This can be due to a variety of reasons were certain unknowns within a complex mixture affect the dissolved hydrogen detection of the palladium‐based SPR sensor.

H_2_S is produced in small quantities in AD and has a negative effect on the sensitivity of palladium to H_2_. Interferences of H_2_S to palladium are partly reversible, but to some extent, these are irreversible 27. This has to be considered when developing a mathematical model for the sensor, as the level of poisoning will have some dynamic trend due to H_2_S poisoning being partly reversible. Sample preprocessing is also possible by significantly increasing the pH of the sample to remove H_2_S; however, this is not desirable as it adds another step to the detection of H_2_ and will probably affect concentrations of other variables within the sample, including H_2_. Development of specific thin film layers on top of the palladium has been shown to reduce the interferences of H_2_S 29.

As with many sensor systems placed inside the liquid phase of an anaerobic digester, biofouling is a real limitation. There are plenty of methods for filtration of samples available that significantly reduce biofouling on the sensor at the cost of filter biofouling. An alternative is to utilize thin non‐stick Teflon films on top of the palladium layer, to limit fouling of the sensor 29. Additionally, by allowing the sensor patch to be replaceable, the sensor could easily be replaced routinely without replacing any of the other components within the sensor setup. Despite this, the selectivity of palladium for H_2_ in such a complex mixture allows many variables within the digestate to be ignored by this sensor.

Optically based SPR sensors integrated directly into the AD would result in rapid online dissolved H_2_ detection within the liquid phase. Direct integration of these sensors (whether directly placed within the reactor, or behind an observation window), and negligible time required to achieve sensor–H_2_ interactions, will allow instantaneous measurements of the dissolved H_2_ within the anaerobic digester. The development of an online sensor system based on SPR sensors for detection of dissolved H_2_ for use in AD, coupled with automated post‐treatment of the sensor data sets is a realistic alternative to traditional H_2_ detection methods. Moreover, multiplexing of sensors along a single optical fiber would allow the combination of further SPR sensors in one optical fiber. For example, the inclusion of sensors to measure CH_4_, NH_4_, and acetic acid alongside dissolved H_2_ in a single optical fiber. This could reduce the requirement for multiple sensors, incorporating the detection of multiple variables into one unit.

## 5 CONCLUDING REMARKS

Current AD plants require sensor advances that permit the estimation of dissolved H_2_ online to avoid inhibition of the biological process, and for future biomethanation development. These advances will give the operators of AD plants the possibility to acquire not only a higher comprehension of the perplexing dynamics inside the AD plant but additionally foresee the heading of the reactor. Therefore, the ability for the described sensor to detect a rapid rise in dissolved H_2_ (irrespective of providing an entirely accurate concentration measurement) would allow rapid alterations to be made to avoid biological inhibition and potential biological process collapse. This will give the controller of the plant valuable time to rectify the biological process, by eliminating the delay in H_2_ detection in the gas phase due to the slow mass transfer of H_2_ between liquid and gas phases.

New sensor frameworks may expect parts to be disposable (e.g. SPR sensor patches); however, they must be able to achieve comparable detection attributes between disposable parts. The lifetime of such sensor segments like the sensor patches might be short in traditional terms; however, must be of low cost so they can be replaced as required. These sensors must be simple, small, and isolated to encourage this. SPR optical‐based sensor advances for liquid phase dissolved H_2_ detection can fulfill the necessities as future online biogas sensors and will fundamentally avoid the identification delay as seen with traditional gas‐phase detection.

## CONFLICT OF INTEREST

The authors have declared no conflict of interest.
